# Microrna-124 targets flotillin-1 to regulate proliferation and migration in breast cancer

**DOI:** 10.1186/1476-4598-12-163

**Published:** 2013-12-13

**Authors:** Laisheng Li, Jinmei Luo, Bo Wang, Dong Wang, Xinhua Xie, Linjing Yuan, Jiaoli Guo, Shaoyan Xi, Jie Gao, Xiaoti Lin, Yanan Kong, Xiangdong Xu, Hailing Tang, Xiaoming Xie, Min Liu

**Affiliations:** 1 Department of Laboratory Medicine, The First Affiliated Hospital of Sun Yat-sen University, Guangzhou 510080, People’s Republic of China; 2 Department of Medical Intensive Care Unit, Third Affiliated Hospital of Sun Yat-sen University, Guangzhou 510630, People’s Republic of China; 3 Department of Breast Oncology, Sun Yat-sen University Cancer Center, Guangzhou 510060, People’s Republic of China; 4 Department of Gynecology, Sun Yat-sen University Cancer Center, Guangzhou 510060, People’s Republic of China; 5 Department of Pathology, Sun Yat-sen University Cancer Center, Guangzhou 510060, People’s Republic of China; 6 Department of Breast Oncology, The First Affiliated Hospital of Sun Yat-sen University, Guangzhou 510080, People’s Republic of China

**Keywords:** Breast cancer, *miR-124*, FLOT1, Proliferation, Migration

## Abstract

**Background:**

MicroRNAs (miRNAs) have been documented as playing important roles in cancer development. In this study, we investigated the role of *miR-124* in breast cancer and clarified the regulation of flotillin-1 (FLOT1) by *miR-124*.

**Methods:**

The expression levels of *miR-124* were examined in breast cancer cell lines and patient specimens using quantitative reverse transcription-PCR. The clinicopathological significance of the resultant data was later analyzed. Next, we explored the function of *miR-124* to determine its potential roles on cancer cell growth and migration *in vitro*. A luciferase reporter assay was conducted to confirm the target gene of *miR-124*, and the results were validated in cell lines and patient specimens.

**Results:**

We found that *miR-124* expression was significantly downregulated in breast cancer cell lines and patient specimen compared with normal cell lines and paired adjacent normal tissues (*P* < 0.0001), respectively. *MiR-124* was also associated with tumor node metastasis (TNM) stage (*P* = 0.0007) and lymph node metastasis (*P* = 0.0004). In breast cancer cell lines, the ectopic expression of *miR-124* inhibited cell growth and migration *in vitro*. Moreover, we identified the FLOT1 gene as a novel direct target of *miR-124*, and *miR-124* ectopic expression significantly inhibited FLOT1. Luciferase assays confirmed that *miR-124* could directly bind to the 3′ untranslated region of FLOT1 and suppress translation. Moreover, FLOT1 was widely upregulated, and inversely correlated with *miR-124* in breast cancer tissues. Consistent with the effect of *miR-124*, the knockdown of FLOT1 significantly inhibited breast cancer cell growth and migration. We also observed that the rescue expression of FLOT1 partially restored the effects of *miR-124*.

**Conclusions:**

Our study demonstrated that *miR-124* might be a tumor suppressor in breast cancer via the regulation of FLOT1. This microRNA could serve as a potential diagnostic marker and therapeutic target for breast cancer.

## Background

Breast cancer is the leading cause of cancer death in females worldwide. Due to the advances in diagnosis and appropriately systemic therapy, including surgery, radiation and chemotherapy, the prognosis of breast cancer is encouraging. However, similar to many other solid tumors, distant metastases account for more than 90% of breast cancer-related death [1]. Because the underlying mechanisms of breast cancer metastasis consist of multiple sequential steps that are not completely understood to date, further investigation of this mechanism is urgently needed.

MicroRNAs (miRNAs) are endogenous noncoding small RNAs that contribute to the regulation of their cognate target genes by usually imperfect base-pairing to the 3′ untranslated region (UTR) of a target mRNA, which results in either mRNA degradation or translation inhibition [2]. In fact, miRNAs are implicated in the regulation of various cellular processes, including proliferation, differentiation, cell death and cell mobility [3]. Furthermore, miRNA profiles also indicate that miRNAs can function either as oncogenes or tumor suppressors in tumor progression [4,5]. Therefore, miRNA expression profiles constitute progress in cancer diagnosis, classification, clinical prognostic information and therapy [6-10].

Previous studies of miRNA profiles demonstrated several deregulated miRNAs in breast cancer, including *miR-124*[11-13]. *MiR-124*, a brain-enriched miRNA, was first found to be involved in stem cell regulation and neurodevelopment [14,15]. Previous research confirmed that *miR-124* is epigenetically silenced in various types of cancer and regulated cancer cell biological behaviors by targeting several important genes, such as sphingosine kinase 1 (SPHK1), rho-kinase2 (ROCK2), enhancer of zeste homologue 2 (EZH2), RAC1, the androgen receptor and CD151 [16-20]. Recent studies further revealed that *miR-124* plays important roles in the regulation of growth, metastasis and epithelial-mesenchymal transition (EMT) in breast cancer [16,21,22]. These studies suggested that *miR-124* can serve as a potential tumor suppressor. Our study showed that *miR-124* was downregulated in breast cancer, and a bioinformatic analysis predicted flotillin-1 (FLOT1) to be a potential target of *miR-124*.

FLOT1 is overexpressed in several types of cancer, including breast cancer [23-26]. FLOT1 was originally identified as a marker of lipids, which is important for non-caveolar raft formation and associated with the development and progression of cancer. In breast cancer, the FLOT1 expression level correlated with clinical staging and prognosis, and its silencing inhibited the proliferation and tumorigenicity of breast cancer cells *in vitro* and *vivo*[26]. MicroRNAs can regulate the expression levels of FLOT1 [27], a process that was intensively studied by our group. Our findings, consistent with other groups, indicated that the role of *miR-124* in the growth and metastasis inhibition was accomplished by the regulation of FLOT1 in breast cancer.

In this study, we aimed to investigate the role of *miR-124* in breast cancer. We found that downregulation of *miR-124* in breast cancer tissues compared with the corresponding normal tissues, and inversely associated with TNM stage and lymph node metastasis in breast cancer. In addition, synthetic *miR-124* mimics inhibited the growth and migration of breast cancer cells *in vitro*. Furthermore, we validated FLOT1, which was overexpressed in breast cancer and predicted as the target of *miR-124*, by 3′-UTR luciferase assays and western blot analysis. Finally, knockdown of FLOT1 consistent with the effects of *miR-124* in breast cancer, and rescue expression of FLOT1 could partially restore these *miR-124* effects. Our study demonstrated that *miR-124* acts as a tumor suppressor by directly targeting FLOT1 in breast cancer, which suggested that *miR-124* has potential diagnostic and therapeutic value for breast cancer treatment.

## Results

### MiR-124 was downregulated in breast cancer cell lines and clinical specimens and inversely associated with advanced clinical stage and lymph node metastasis

**Figure 1:**
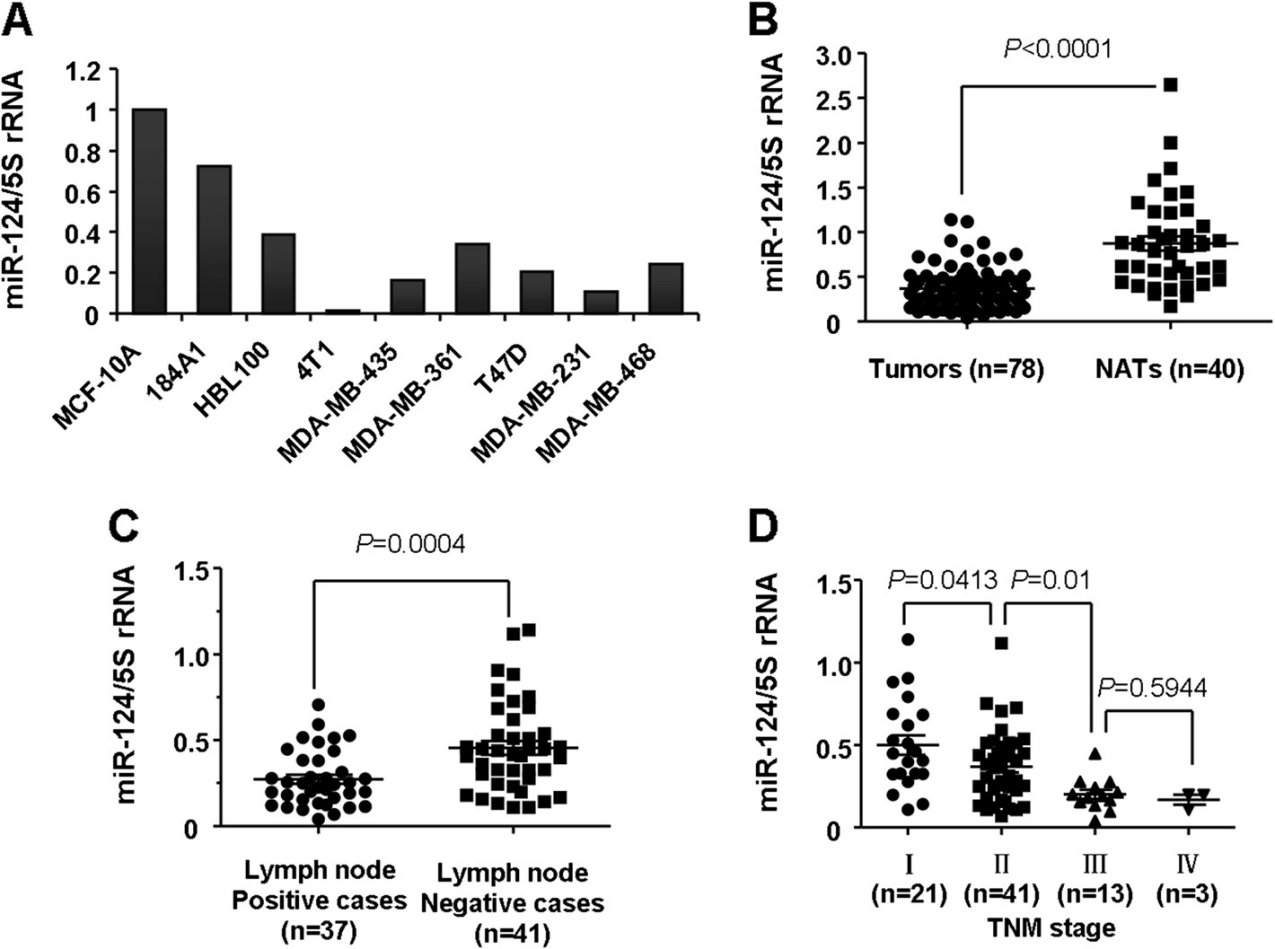
***miR-124 *is downregulated in breast cancer cell lines and human breast cancer specimens, and the reduced expression is associated with advanced clinical stage and positive lymph node metastasis in breast cancer patients. A**, Expression of *miR-124* in 7 breast cancer cell lines and 2 normal breast epithelial cell lines; *miR-124* expression was determined by qRT-PCR and normalized to 5S rRNA. **B**, Expression of *miR-124* in 78 cases of human breast cancer and 40 corresponding normal adjacent tissues. **C**, Expression levels of *miR-124* are associated with lymph node metastasis of breast cancer (*P* = 0.0004). **D**, Expression levels of *miR-124* in different clinical stages of breast cancer patients (*P* = 0.0004). Statistical analysis is performed using the paired t-test **(B)** and the Student t-test **(C and D)**. Data are shown as the mean ± SEM. NATs: normal adjacent tissues.

To study the expression level of *miR-124* in breast cancer, a panel of breast cancer cell lines was first analyzed by stem-loop RT-PCR. Compared with the two immortalized normal mammary epithelial cell lines (184A1, MCF-10A), *miR-124* expression level was downregulated in all 7 breast cancer cell lines (MDA-MB-231, MDA-MB-361, MDA-MB-435, MDA-MB-468, MCF-7, HBL100, T47D, 4 T1) (Figure 1A).

We further assessed the expression levels of *miR-124* in 78 clinical human primary breast cancer tissues and 40 paired normal adjacent tissues (NATs) to analyze the clinicopathologic significance of the *miR-124*. The relationship between the *miR-124* expression levels and clinicopathologic characteristics in breast cancer patients are summarized in Table 1. Consistent with the result obtained from breast cancer cell lines, the average expression level of *miR-124* was downregulated in breast cancer tissues compared with paired normal adjacent tissues (Figure 1B; *P* < 0.0001). We divided 78 breast cancer cases into two groups according to the status of lymph node metastasis: lymphatic node metastasis positive or negative. Interestingly, the breast cancer lymphatic node metastasis positive group (n = 37) showed an even lower *miR-124* expression level than the lymphatic node metastasis negative group (n = 41; Figure 1C; *P* = 0.0004). In addition, we also found that the expression of *miR-124* was lower in advanced TNM stage breast cancer patients (stage III and IV) than early stage patients (stage I and II; Figure 1D and Table 1). Taken together, these results indicated that *miR-124* is downregulated in breast cancer, and a reduced expression of *miR-124* may play an important role in the progression and metastasis of breast cancer.

**Table 1:** **The relationship between *miR-124 *** **expression and clinicopathological parameters in breast cancer**

**Clinicopathologic parameters**	**Number of cases**	**Median expression of miR-124**	***P*-value**
**Age (year)**			
≤45	38	0.4252 ± 0.0403	0.0372
>45	40	0.3140 ± 0.0338	
**Tumor size (cm)**			
≤2	25	0.4107 ± 0.0573	0.2781
>2	53	0.3481 ± 0.0287	
**TNM stage**			
I + II	62	0.4128 ± 0.0307	0.0007
III + IV	16	0.1951 ± 0.0231	
**Lymph node metastasis**			
No	41	0.4550 ± 0.0407	0.0004
Yes	37	0.2720 ± 0.0265	
**ER status**			
Negative	25	0.3575 ± 0.0495	0.7861
Positive	53	0.3732 ± 0.0320	
**PR status**			
Negative	31	0.3426 ± 0.0395	0.4423
Positive	47	0.3850 ± 0.0361	
**Her2 status**			
Negative	49	0.3548 ± 0.0326	0.5221
Positive	29	0.3906 ± 0.0469	

### Ectopic expression of miR-124 inhibited the proliferation, migration and invasion of breast cancer cells

**Figure 2:**
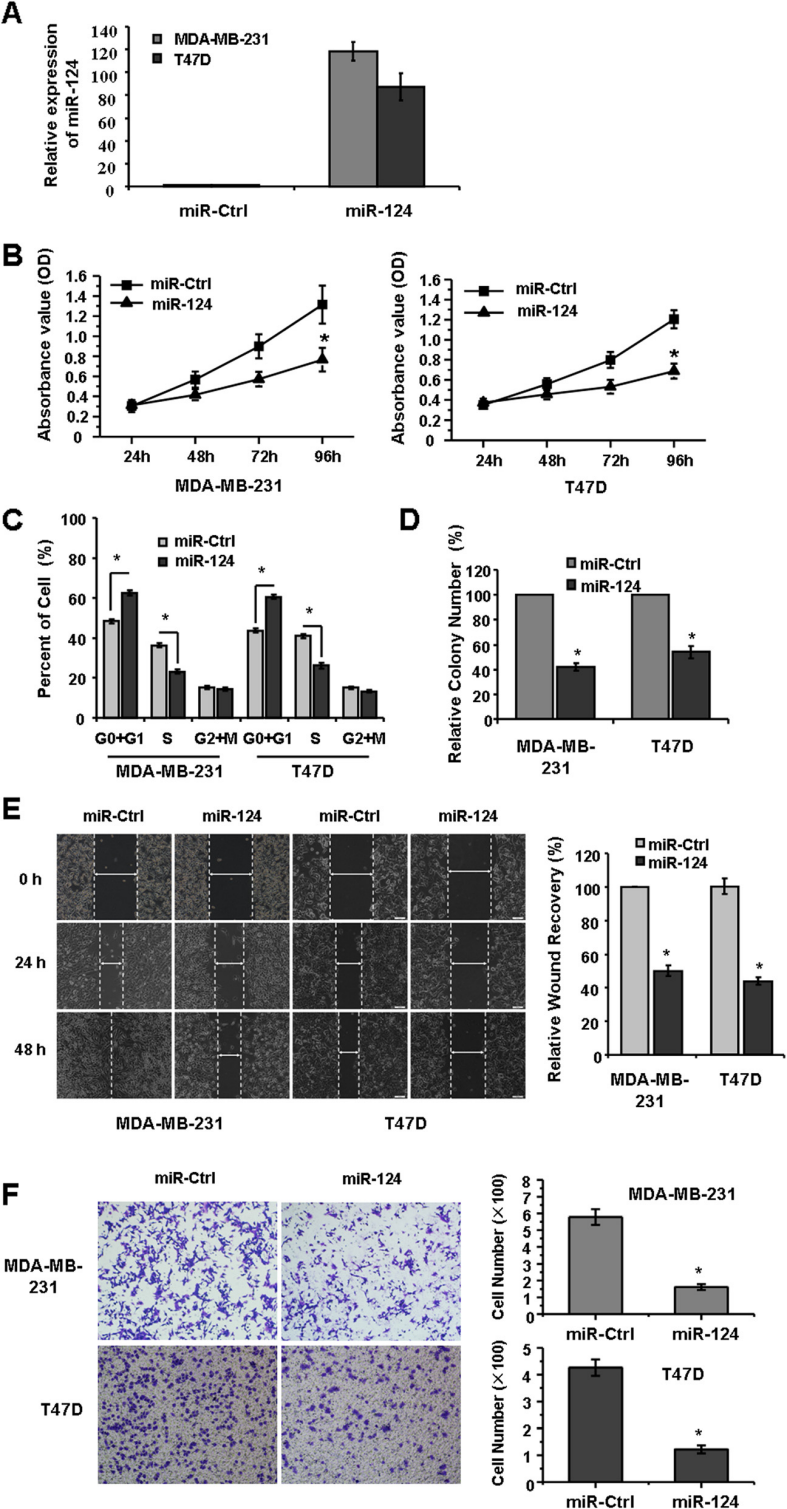
**Ectopic expression of *miR-124 *suppresses breast cancer cell proliferation, migration and invasion. A**, *miR-124* was re-expressed in MDA-MB-231 and T47D cells by transfection of *miR-124* mimics. Forty-eight hours later, the *miR-124* expression levels were tested by quantitative RT-PCR and normalized to 5S rRNA. **B**, Analysis of the effect of *miR-124* on the proliferation of MDA-MB-231 and T47D cells by MTT assay. **C**, Cell cycle arrest effect of *miR-124* on MDA-MB-231 and T47D cells monitored with flow cytometric. **D**, Colony formation assay demonstrated that the effect of *miR-124* on the cell growth of MDA-MB-231 and T47D cells. **E**, Analysis the effect of *miR-124* on the migration of MDA-MB-231 and T47D cells by wound-healing assay. **F**, A Matrigel invasion chamber was utilized to analyze the effect of *miR-124* on the cell invasion of MDA-MB-231 and T47D cells. The data represent the mean values of three independent experiments. *, *P* < 0.05 with paired *t-*test.

To investigate the effect of *miR-124* on cell proliferation, we transfected the breast cancer cell lines MDA-MB-231 and T47D with *miR-124* mimics. The successful overexpression of *miR-124* in the cells was confirmed by quantitative real-time PCR (Figure 2A). MTT and colony formation assays showed that ectopic expression of *miR-124* could markedly inhibit the proliferation and growth of MDA-MB-231 and T47D cells compared with the mimic control (Figure 2B and D; *P* < 0.05). This anti-proliferation effect could be partially due to the disruption of cell growth regulation, such as cell cycle arrest. Thus, we next explored the effect of *miR-124* on cell cycle regulation. Flow cytometric cell cycle analysis showed that *miR-124* increased the number of cells in the G0 + G1 phase and decreased the number of cells in the S and G2 + M phase in the MDA-MB-231 and T47D breast cancer cell lines compared with miR-Ctrl (Figure 2C).

After confirming the cell proliferation and growth inhibition ability of *miR-124*, we investigated the role of *miR-124* in cell migration and invasion. Wound healing and Matrigel invasion assays demonstrated that the ectopic expression of *miR-124* inhibited the cell migration and invasion of MDA-MB-231 and T47D cells compared with the mimic control (Figure 2E and F). The above results support the role of *miR-124* in the inhibition of breast cancer proliferation, migration and invasion and suggest that *miR-124* has a tumor suppressor function.

### MiR-124 downregulated FLOT1 expression by directly targeting its 3′-UTR

**Figure 3:**
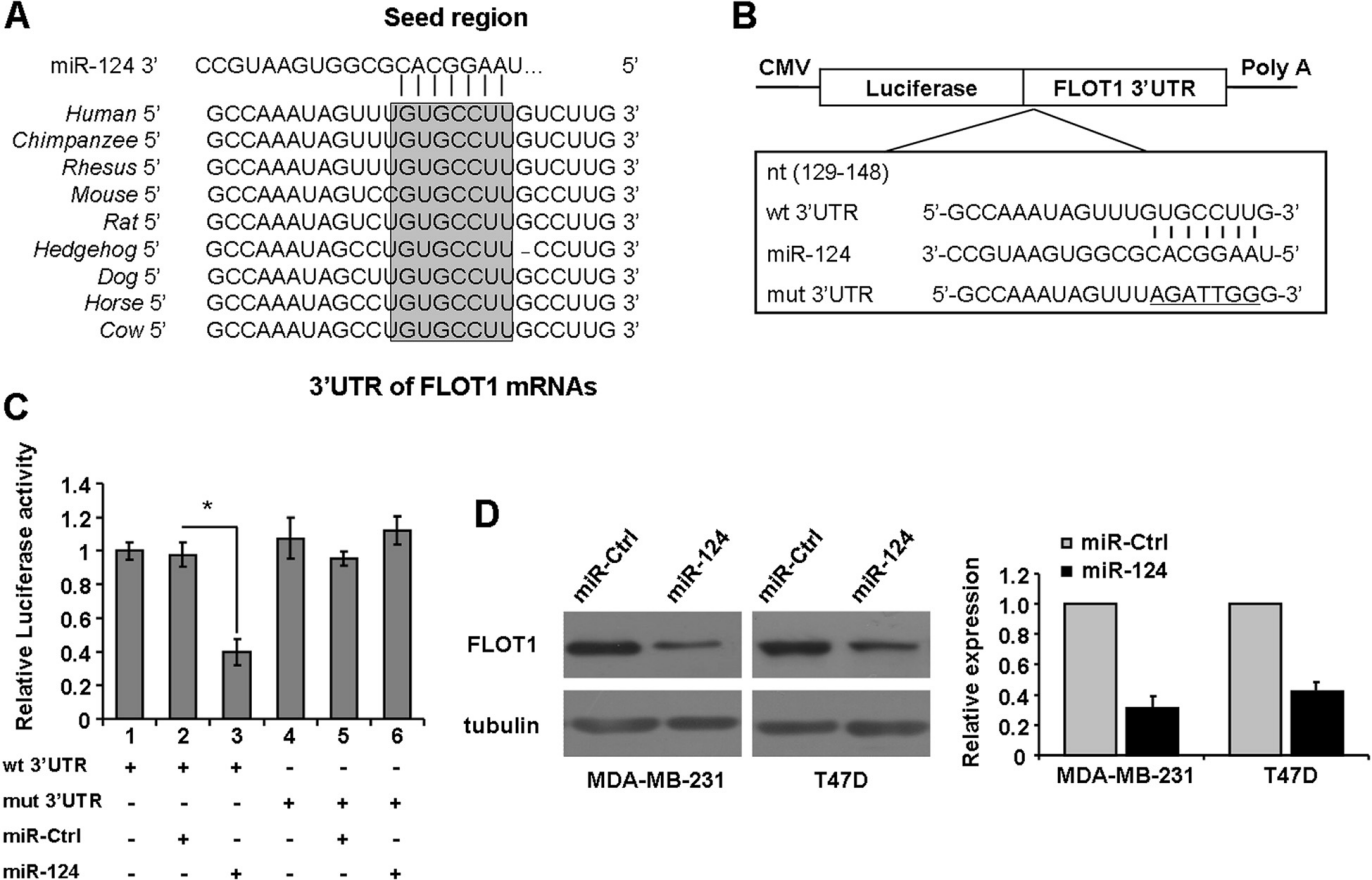
***MiR-124 *directly regulates FLOT1 by binding to the FLOT1 3′-UTR. A**, Putative *miR-124* binding site in the 3′-UTR region of FLOT1 and interspecies conservation of seed matching sequences (gray box). **B**, Diagram of FLOT1 3′-UTR contained reporter constructs. **C**, Luciferase reporter assays in MDA-MB-231 cells. Co-transfected of wt/mut 3′-UTR with miRNAs as indicated. **D**, Expression levels of FLOT1 are tested after *miR-124* transfected at 50 nM in MDA-MB-231 and T47D by western blot assay. *, *P* < 0.05 compared with control.

To elucidate the molecular mechanism responsible for the proliferation and migration inhibition induced by *miR-124* in breast cancer cells, we used a bioinformatic analysis to search for putative protein-coding gene targets of *miR-124*, especially for those that can promote cancer cell growth and metastasis. According to this rationale, FLOT1 was selected as one of the candidate targets of *miR-124*, which was highly conserved among different species and whose 3′-UTR of mRNA contained a complementary site for the seed region of *miR-124* (Figure 3A).

We performed a luciferase reporter assay to determine whether FLOT1 is a direct target of *miR-124* in breast cancer cells. The target region sequence of FLOT1 3′-UTR (wt 3′-UTR) or the mutant sequence (mut 3′-UTR) was cloned into a luciferase reporter vector (Figure 3B). These constructed reporter vectors were co-transfected with *miR-124* mimics or miR-Ctrl into the MDA-MB-231 cell line. The data in Figure 3C show that *miR-124* could downregulate the luciferase activity of the FLOT1 wt 3′-UTR construct (Figure 3C, lanes 3; *P* < 0.05), whereas the luciferase activity was not significantly attenuated in the target region of the mutated mut 3′-UTR construct (Figure 3C, lanes 4, 5 and 6). These data suggest that the regulation of *miR-124* on FLOT1 depended on the specific seed region sequence. Moreover, miR-Ctrl did not significantly affect the luciferase activity of either the wt or mut 3′-UTR construct (Figure 3C, lanes 2 and 5).

We further analyzed the FLOT1 protein expression by using western blotting after transfecting MDA-MB-231 and T47D cells with *miR-124* mimics. As shown in Figure 3D, the ectopic expression of *miR-124* inhibited FLOT1 expression by approximately 60% to 70%. Therefore, we concluded that *miR-124* inhibited FLOT1 expression by binding to the 3′-UTR sequences of FLOT1 in breast cancer.

### Knockdown of FLOT1 induced inhibition of breast cancer cells proliferation and invasion

**Figure 4:**
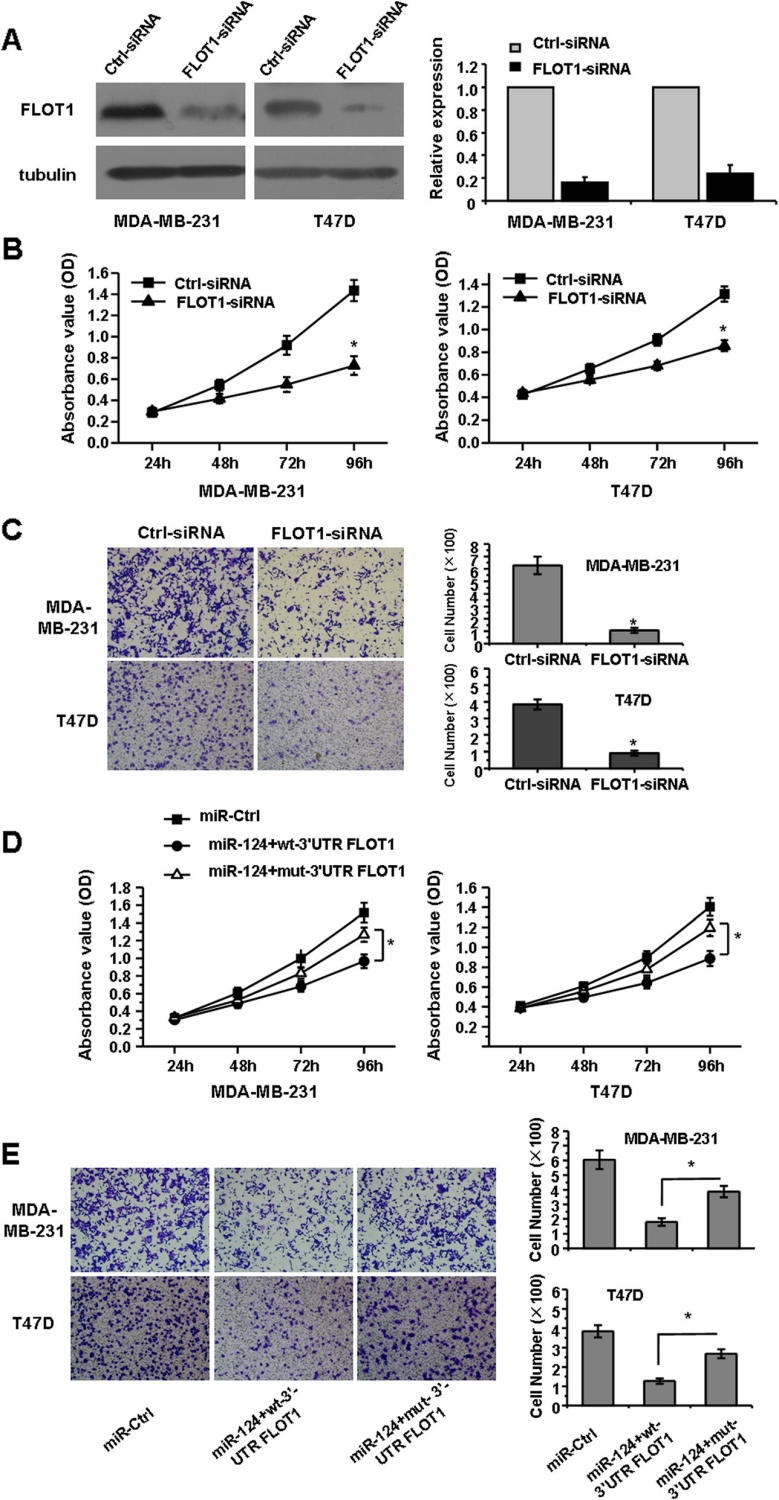
**FLOT1 is essential for breast cancer cell proliferation and invasion, and involved in the *miR-124*-induced inhibition of cell proliferation and migration. A**, Analysis of protein expression by western blot, FLOT1 protein is reduced by small interfering RNA (FLOT1-siRNA) in MDA-MB-231 and T47D cells. **B**, MTT assay show that cell proliferation is inhibited in MDA-MB-231 and T47D cells after transfection of FLOT1-siRNA compared with scrambled sequence (Ctrl-siRNA). **C**, Cell invasion of MDA-MB-231 and T47D cells after knockdown of FLOT1 are evaluated by Matrigel invasion chamber. **D**, MTT assay shows cell proliferation in MDA-MB-231 and T47D cells co-transfected with *miR-124* and wt/mut 3′-UTR-FLOT1 compared with scrambled miRNA mimics (miR-Ctrl). E, Cell invasion ability of MDA-MB-231 and T47D cells which co-transfection of *miR-124* and wt/mut 3′-UTR-FLOT1 were evaluated by Matrigel invasion chamber compared with scrambled miRNA mimics (miR-Ctrl). *, *P* < 0.05 compared with control.

To explore the function of FLOT1 in breast cancer, MDA-MB-231 and T47D cells were transfected with FLOT1-specific siRNAs (FLOT1-siRNA). A western blot analysis indicated that FLOT1 protein decreased significantly after 48 hours in both MDA-MB-231 and T47D cells transfected with 50 nM FLOT1-siRNA (Figure 4A). The MTT and colony formation assays showed that the knockdown of FLOT1 inhibited the proliferation and growth of both MDA-MB-231 and T47D cells (Figure 4B; *P* < 0.05). Furthermore, a Matrigel invasion assay indicated that the knockdown of FLOT1 inhibited the invasion of breast cancer cells (Figure 4C), an effect that resembled the inhibitory effect of *miR-124* in breast cancer cells.

To test whether FLOT1 is the direct functional mediator of the *miR-124* induced inhibition of breast cancer cell proliferation and migration, we co-transfected *miR-124* mimics along with wt/mut 3′-UTR-FLOT1 plasmid which FLOT1 cDNA contained wild type or mutant 3′ UTR into breast cancer cells. MTT and Matrigel invasion assays showed that mut 3′-UTR-FLOT1 could partially abrogate the *miR-124*-mediated effects in breast cancer cells, to restore the proliferation and migration compared with the miR-Ctrl (Figure 4D and E; *P* < 0.05). Therefore, FLOT1 has an important role in the proliferation and invasion of breast cancer cells, which was regulated by *miR-124*.

### MiR-124 and FLOT1 are inversely correlated in breast cancer tissues

**Figure 5:**
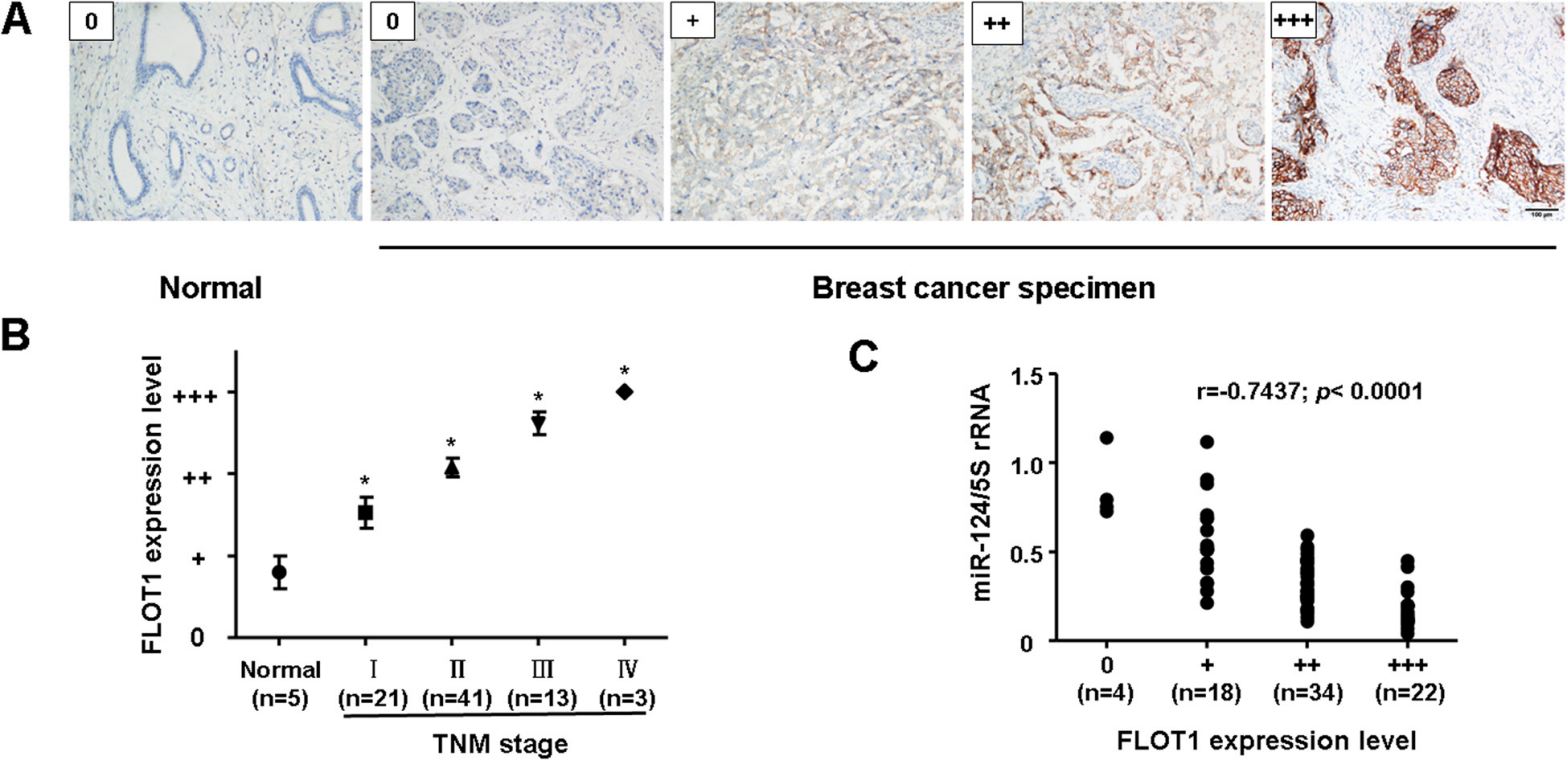
***MiR-124 *and FLOT1 are inversely correlated in breast cancer tissues. A**, Representative IHC analysis of FLOT1 in normal breast tissues, and breast cancer specimen with different clinical stages. **B**, Statistical quantification of the IHC scores of FLOT1 between normal breast tissues and breast cancer specimen with different clinical stages. **C**, A scatter diagram shows an inverse correlation between *miR-124* and FLOT1 expression in the same set of breast cancer tissue (Spearman’s correlation analysis, r = −7437; *P* < 0.0001).

IHC was performed to detect FLOT1 expression in 5 normal breast tissues and 78 clinical breast cancer specimens, and the *miR-124* expression levels were simultaneously analyzed by RT-PCR. These cases included 21 cases of clinical stage I, 41 cases of stage II, 13 cases of stage III, and 3 cases of stage IV breast cancers. FLOT1 was found to be predominantly overexpressed in the cytoplasm and membranes of breast cancer tumor cells and was less expressed in adjacent normal tissues (Figure 5A). In consensus with previous report [26], our data also showed that FLOT1 expression in stage I to IV primary tumors was statistically higher than in normal breast tissues (*P* < 0.05, Figure 5B). We then correlated FLOT1 with the *miR-124* expression levels in the same breast cancer specimens. As shown in Figure 5C, a significant inverse correlation was observed when FLOT1 expression levels were plotted against *miR-124* expression levels (2-tailed Spearman’s correlation, r = −07437; *P* <0.0001).

## Discussion

Cancer is characterized by abnormal and uncontrolled cell proliferation, which is caused not only by the misregulation of several pivotal proteins but also by a systemic change in the miRNAs profile [28]. MiRNAs are involved in the regulation of multiple biological processes, including development, cell proliferation, apoptosis, differentiation, disease survival and cell death [29,30]. Considering the function of miRNAs, their deregulation expectedly contributes to substantial cell physiological and pathological processes and is ultimately involved in tumorigenesis and the tumor progression of many different human cancers. In this report, we showed that *miR-124* was markedly downregulated in human breast cancer cell lines and clinical specimens compared with immortalized normal mammary epithelial cell lines and normal adjacent tissues, respectively. *MiR-124* downregulation was significantly associated with advanced clinical stage and positive lymph node-metastasis in breast cancer patients. Furthermore, the ectopic expression of *miR-124* inhibited breast cancer cell proliferation, migration and invasion. Moreover, FLOT1 was identified as a direct and functional target of *miR-124* via binding to the 3′UTR of FLOT1. Our study suggested that *miR-124* acts as a novel proliferation and metastasis suppressor in breast cancer, and downregulated *miR-124* contributes to lymph node-metastasis and tumor progression in breast cancer patients.

Although *miR-124* was identified long ago, its biological function has only recently been investigated. *MiR-124* acts as a tumor suppressor, and its downregulation has been identified in various cancers [16,18-22,31-33], which suggests that *miR-124* may play a vital role in tumorigenesis and tumor progression. *Shi et al.* showed that *miR-124* was a potential tumor-suppressive miRNA and was downregulated in prostate cancer to result in proliferation inhibition of prostate cancer cells by targeting the androgen receptor [20]. *Wang et al.* reported that *miR-124* was epigenetically silenced in pancreatic cancer and inhibited cell proliferation and metastasis by regulating Rac1 [17]. *Zheng et al.* showed that *miR-124* levels were frequently reduced in hepatocellular carcinoma, and this expression level was significantly associated with the patients’ clinical stages and prognoses and regulated the invasion and migration of hepatocellular carcinoma through post-transcriptional regulation of ROCK2 and EZH2 [19]. *Lv et al.*, *Liang et al.* and *Han et al.* also reported that *miR-124* can suppress breast cancer growth and metastasis [16,21,22]. *Han et al.* found that *miR-124* is downregulated in breast cancer and the ectopic expression of *miR-124* could suppress the invasion and metastatic ability, likely by directly targeting the CD151. CD151 regulates the ligand biding activity of integrin α3β1 and plays a role in Met-dependent signaling and TGF-β signaling, while c-met can regulate many cellular process, especially the proliferation and migration of cancer. These results suggest an important role for *miR-124* in the proliferation and metastasis of different cancers. However, the *miR-124* expression levels in clinical specimens and its exact mechanism in breast cancer has not been clearly elucidated. In this study, we demonstrated for the first time that *miR-124* was frequently downregulated in breast cancer, and the average expression levels of *miR-124* were significantly downregulated in breast cancer tissues compared with paired normal adjacent tissues. Interestingly, we found that lower levels of *miR-124* are associated with advanced TNM stage (stage I + II vs. stage III + IV, P = 0.0007) and positive lymph node metastasis, suggesting that a low expression of *miR-124* is associated with breast cancer progression. Recently, *miR-124* was reported to be subject to epigenetic regulation in various tumors, including breast cancer [17,31,32,34], which in turn may explain the downregulation of *miR-124* in breast cancer. Taken together, these results suggest that *miR-124* expression is frequently reduced in breast cancer, which may be responsible for the tumorigenesis and progression of breast cancer. However, the function of *miR-124* in breast cancer is not fully understood.

The capability of cells to proliferate, migrate and invade is considered an important determinant in the process of tumorigenesis and progression. Many oncogenes and suppressor genes reportedly correlate with the course of cancer initiation and progression, but the molecular mechanisms are not fully understood. Recently, accumulating studies have reported that miRNAs play important roles in breast cancer tumorigenesis and progression [35-37]. Interestingly, a number of miRNAs are associated with the proliferation and migration of breast cancer, such as *miR-26a*[38], *miR-34a*[39], *miR-137*[40], and *miR-210*[41], which may provide new insights into the design of eradicating therapeutic strategies for breast cancer. Even *miR-124* has been reported as a tumor suppressor miRNA in breast cancer. However, the mechanisms involved have not been fully elucidated. To determine the function of *miR-124* in breast cancer, we tested the effect of *miR-124* on MDA-MB-231 and T47D cell lines. Ours results indicate that *miR-124* could suppress breast cancer cell proliferation, migration and invasion, which suggests its role as a tumor suppressor in breast cancer.

In the current study, we identified FLOT1 as a direct and functional target of *miR-124*. The protein encoded by the FLOT1 gene is an integral membrane protein that participates in vesicular trafficking, signal transduction and is important for lipid raft formation [25,26,42]. Accumulating evidence shows that the overexpression of FLOT1 in various cancers contributes to proliferative and invasive behavior as well as a worse prognosis [24-26]. The knockdown of FLOT1 reportedly suppressed the proliferation and tumorigenesis of breast cancer cells by enhancing the transcriptional activity of FOXO3a, inhibiting Akt activity, downregulating cyclin D1 and upregulating the cyclin-dependent kinase inhibitors p21^Cip1^ and p27^Kip1^[26]. *Xiong et al.* also showed that Flotillin-1 could clearly activate the growth and metastasis of oral squamous carcinoma by transfecting cells with a Flotillin-1 expression vector or shRNA targeted Flotillin-1. This effect was mediated by the activation of the NF-κB signaling pathway, which enhanced the phosphorylation of p65 and IκBα [23]. These studies showed that FLOT1 can regulate many cellular processes, particularly in cancer growth, proliferation, migration, metastasis and tumorigenesis. Consistent with the study above, we found that *miR-124* could directly target and downregulate FLOT1, and high FLOT1 expression was associated with low *miR-124* levels in breast cancer specimens. These findings provide new insight into the essential mechanisms of FLOT1 regulation in breast cancer. Additionally, *miR-138* was also reported to regulate FLOT1 in esophageal squamous cell carcinoma. These findings suggest that the post-transcriptional regulation of FLOT1 by miRNAs is a vital mechanism underlying cancer proliferation and metastasis, and *miR-124* may serve as potential treatment target for regulating FLOT1 to inhibit the growth and metastasis of breast cancer.

## Conclusions

Our study demonstrates that *miR-124* is downregulated and inversely associated with the lymph node metastasis in breast cancer. The ectopic expression of *miR-124* inhibits cell proliferation and migration by downregulating FLOT1, which indicates the internal mechanism of tumor suppression of *miR-124*. Combined with the above mentioned studies, this work contributes to the understanding of the effect of *miR-124* on tumor suppression. This study suggests that *miR-124* downregulated may play an important role in tumor proliferation and migration and may be a novel diagnostic marker and potential therapeutic target in breast cancer.

## Materials and methods

### Human breast cancer tissues

78 cases of human breast cancer and 40 corresponding normal breast tissues were collected at the time of surgical resection from the First Affiliated Hospital of Sun Yat-sen University and Sun Yat-sen University Cancer Center from (Guangzhou, China) 2009 to 2011. The samples were fixed in RNAlater (Ambion, Austin, TX, USA) immediately after surgical resection and stored at −80°C in a freezer until use. The breast cancer samples selected were based on a clear pathological diagnosis, and the clinical information for the samples is presented in Table 1. The tumor stage was defined according to the American Joint Committee on Cancer and tumor-lymph node-metastasis classification system [43]. All patients provided consent for the use of their specimens in research, and this use was approved by the institute research ethics committee of the First Hospital of Sun Yat-sen University.

### Immunohistochemical staining

Inmmunohistochemistry (IHC) staining of formalin-fixed and paraffin-embedded tissue slides was performed and quantified as previously described [44]. Briefly, 5 μm tissue slides were deparaffinized, rehydrated via a series of descending graded alcohols and subjected to antigen retrieval in 0.01 M citrate buffer (pH 6.0) at 90°C for 40 minutes. Following a blocking step, the slides were incubated with FLOT1 primary antibody (1:500; Sigma, Saint Louis, MO) and washed. Biotinylated secondary antibody was applied, and the immunocomplexes were visualized using an avidin-biotin complex immunoperoxidase system (Vector Laboratories, Burlingame, CA, USA) with 0.03% diaminobenzidine (DAB) as a chromagen and hematoxylin as the counterstain. We used phosphate-buffered saline (PBS) instead of the primary antibody as a negative control, and a composite slide containing formalin-fixed cell pellets of MDA-MB-231 and T47D as positive control to assess the quality of the IHC reaction. The slides were reviewed and scored independently based on both the percentage of positive stained tumor cells and overall stained intensity by two observers who were blinded to specimens’ clinical information. The following scoring rubric was used: scored 0, absent positive tumor cells; scored +, weak cell staining or <10% positive tumor cells; scored ++, moderate cell staining or 10-50% positive tumor cells; scored +++, strong cell staining or >50% positive tumor cells. Conflicts (approximately 5% of cases) were resolved by consensus.

### Cell culture

The breast cancer cell lines MDA-MB-231, MDA-MB-361, MDA-MB-435, MDA-MB-468, MCF-7, HBL100, T47D, and 4 T1 and two immortalized normal mammary epithelial cell lines, MCF-10A and 184A1, were obtained from the American Type Culture Collection (Manassas, VA) and freshly recovered from liquid nitrogen (<3 months). The breast cancer cells were maintained according to the vendor’s instructions. Briefly, the breast cancer cells were maintained in Dulbecco’s modified Eagle’s medium (DMEM) or RPMI 1640 (Invitrogen, Beijing, China) supplemented with 10% fetal bovine serum (FBS, GIBCO, Cappinas, Brazil). MCF-10A cells were cultured in Keratinocyte-SFM (Invitrogen, CA, USA) supplemented with pre-qualified human recombinant epidermal growth factor 1–53 (EGF 1–53, Invitrogen, CA, USA) and bovine pituitary extract (BPE, Invitrogen, CA, USA). The 184A1 cells were cultured in Mammary Epithelium Basal Medium (MEBM, Clonetics, MD, USA). All cells were grown and maintained at 37°C in a 5% CO_2_ humidified incubator (Thermo Electron Corp, New Castle, DE).

### Bioinformatics

The analysis of *miR-124* predicted targets was determined using the algorithms of TargetScan 5.1 (http://www.targetscan.org/) and miRanda (http://www.microrna.org). According to these algorithms, we predicted that the FLOT1 gene might be a direct target of miR-124.

### Transient transfection of miRNA and siRNA

The *miR-124* mimics, a non-specific miRNA negative control (miR-Ctrl), small interfering RNA (siRNA) duplexes targeting human FLOT1 (FLOT1-siRNA) (sense strand, 5′-ACAGAGAGAUUACGAACUGAAdTdT-3′ and antisense strand, 5′-UUCAGUUCGUAAUCUCUCUGUdTdT-3′) and scrambled control siRNA (Ctrl-siRNA) (sense strand, 5′-UUCUCCGAACGUGUCACGUdTdT-3′ and antisense strand, 5′-ACGUGACACGUUCGGAGAAdTdT-3′) were synthesized and purified by RiboBio (Guangzhou, China). MiRNA mimics or siRNA duplexes were transfected at working concentrations of 50 nM using Lipofectamine 2000 reagent (Invitrogen, CA, USA), according to the manufacturer’s instruction.

### RNA extraction and quantitative real-time PCR

The RNA extraction and quantitative real-time PCR procedure were carried out as previously reported [39]. Briefly, total RNA was extracted using TRIzol® Reagent (Invitrogen, CA, USA). To quantitate the *miR-124* expression, reverse transcription was performed with a specific stem-loop real-time PCR miRNA kit (RiboBio, Guangzhou, China). Quantitative real-time PCR (qPCR) was performed using the Platinum SYBR Green qPCR SuperMix-UDG system (Invitrogen, CA, USA) on an Applied Biosystems 7900HT real-time PCR system, and the data were collected and analyzed using ABI SDS version 2.3. All procedures were performed according to the manufacturer’s instructions. 5S rRNA was used as an internal control. All samples were normalized to internal controls, and the fold changes were calculated according to the relative quantification method (RQ = 2^−ΔΔCT^). The results are shown as fold changes of expression in cells or cancer tissues.

The primers of *miR-124* and 5S rRNA used for stem-loop real-time PCR are listed as follows: *miR-124* stem-loop RT, 5′-GTCGTATCCAGTGCAGGGTCCGAGGTATTCGCACTGGATACGACATCAAG-3′; miR-124 forward, 5′-GCGGCCGTGTTCACAGCGGACC-3′; miR-124 reverse, 5′-GTGCAGGGTCCGAGGT-3′; 5S rRNA stem-loop RT, 5′-GTCGTATCCAGTGCAGGGTCCGAGGTATTCGCACTGGATACGACCAGGCG-3′; 5S rRNA forward, 5′-CTGGTTAGTACTTGGACGGGAGAC-3′; 5S rRNA reverse, 5′-GTGCAGGGTCCGAGGT-3′.

### MTT assay

The cell viability and proliferation of MDA-MB-231 and T47D with miRNA mimics or siRNA duplexes were determined by 3-(4, 5-dimethylthiazolyl-2-yl)-2-5 diphenyl tetrazolium bromide (MTT, Sigma, St. Louis, MO, USA) assay. The cells were plated in 96-well plates at 5 × 10^3^ per well in a final volume of 100 μL and treated with miRNA mimics or siRNA duplexes. After incubation for 24, 48, 72 and 96 hours, the culture medium was replaced with 100 μL of fresh DMEM. Twenty-five microliters of MTT stock solution (5 g L^-1^ in phosphate-buffered saline) were added to each well to achieve a final concentration of 1 g L^-1^. The plates were incubated for another 4 hours, the culture medium was replaced with dimethyl sulfoxide (DMSO, Sigma, St. Louis, MO, USA), and the absorbance was measured at 570 nm by a SpectraMax M5 Microplate Reader (Molecular Deviced, Sunnyvale, CA, USA). The cell viability was normalized to that of cells cultured in the culture medium without miRNA mimics or siRNA duplexes. Three independent experiments (3 replicates in each) were performed.

### Wound healing assay

To determine cell migration, MDA-MB-231 and T47D breast cancer cells transfected with miRNA mimics were seeded in six-well plates, incubated in their respective complete culture medium and grown to confluence overnight. Wounds were made by scraping with a sterilized 200 μL pipette tip, and the debris was rinsed with phosphate-buffered saline. Serial photographs were obtained at 0, 24 and 48 hours using a phase contrast microscope (Olympus IX81, Tokyo, Japan).

MiRNA-transfected cells were scratched using a standard 200 μL tip. The debris was removed by washing with serum-free medium. Serial photographs were obtained at different time points using a phase contrast microscope (Olympus IX81, Tokyo, Japan). Three independent experiments were carried out.

### Transwell invasion assays

To determine cell invasion *in vitro*, Matrigel-coated invasion chambers (8 μm; BD Biosciences, CA, USA) were used according to the manufacturer’s protocol. Briefly, miRNA mimic- or siRNA duplex-transfected cells were harvested, re-suspended (1 × 10^5^ cells per well) in 200 μL serum-free medium, and transferred to the upper chamber of the Matrigel-coated inserts; culture medium containing 10% FBS was placed in the bottom chamber. The cells were incubated for 24 hours at 37°C; the cells on the upper surface were then removed by peeling off the matrigel and swiping the top of the membrane with cotton swabs. The cells that had invaded the lower surface were fixed and stained with 0.5% crystal violet (Sigma, St Louis, MO, USA) for 30 min, counted under an inverted microscope (Olympus IX71, Tokyo, Japan), and the relative number of invading cells was calculated from five-field digital images taken randomly at 200× magnification. The data are the means ± SD of three independent experiments.

### Cell cycle assays

To determine cell cycle distribution, the cells were plated in 6-well plates and transfected with miRNA mimics or siRNA duplexes. After transfection, the cells were collected by trypsinization, fixed in 70% ethanol, washed in PBS, re-suspended in 200 ml of PBS containing 1 mg/ml RNase, 0.05% Triton X-100 and 50 mg/ml propidium iodide (Sigma, St Louis, MO, USA), incubated for 30 min at 37°C in the dark, and analyzed immediately using a FACSCalibur instrument (Becton Dickinson, CA, USA). The data were analyzed using the CellQuest Pro software (BD Biosciences).

### Colony formation assays

After transfecting with miRNA mimics or siRNA duplexes, the cells were seeded in 6-well plates at 5 × 10^2^ per well and incubated for 2 weeks for the colony formation assay.

The cells were then washed twice with PBS, fixed with methanol/acetic acid (3:1, v/v), and stained with 0.5% crystal violet (Sigma, St Louis, MO, USA). The number of colonies was counted under the microscope (Olympus IX81, Tokyo, Japan).

### Plasmid

The 3′-untranslated regions (3′-UTR) sequences of human FLOT1 containing the putative *miR-124* binding sites were isolated from MDA-MB-231 cDNA using PCR amplification and cloned into the pGL3 vector (Promega, Madison, WI, USA), which was termed as wild-type 3′-UTR (wt 3′-UTR). The point mutations in the putative *miR-124* binding seed regions were performed using the Quick-Change Site-Directed Mutagenesis kit (Stratagene, La Jolla, CA, USA) according to the manufacturer’s protocol. The resultant product served as the mutated 3′-UTR (mut 3′-UTR). Both the wild-type and mutant insert fragments sequences were confirmed by DNA sequencing.

For FLOT1 overexpression, the cDNA of FLOT1 containing the putative *miR-124* binding sites was cloned into the multiple cloning site of the pcDNA3.1 vector (Invitrogen, Carlsbad, CA, USA), which was termed as wild-type 3′-UTR-FLOT1 (wt 3′-UTR-FLOT1). The mut 3′-UTR-FLOT1 was obtained as described above. In the rescue experiment, cells were cotransfected with 50 nM of miRNA mimics and 500 ng of plasmid in a six-well plate.

### Luciferase assays

The cells were seeded in triplicate in 24-well plates one day before transfection for the luciferase assays. Wt or mut 3′-UTR vectors and the control vector pRL-TK (Promega, Madison, WI, USA) coding for Renilla lucifearse were co-transfected with *miR-124* mimics or negative control into MDA-MB-231 cells using Lipofectamine 2000 reagent, as described previously. After 48 hours of transfection, the cells were harvested and lysed, and the luciferase activity was assayed using the Dual-Glo luciferase assays kit (Promega, Madison, WI, USA). The firefly luciferase values were normalized to Renilla, and the relative ratios of firefly to Renilla activity were reported. Three independent experiments were performed, and the data are presented as the mean ± SD.

### Western blot analysis

Transfected MDA-MB-231 and T47D cells were cultured for 72 hours and then harvested on ice using RIPA lysis and extraction buffer (25 mM Tris–HCl pH 7.6, 150 mM NaCl, 1% NP-40, 1% sodium deoxycholate, 0.1% SDS, protease inhibitor cocktail (Pierce, Rockford, IL). The total cell extracts (20 μg protein) were separated using 10% SDS-polyacrylamide gels and electrophoretically transferred to polyvinylidene difluoride membranes (PVDF, Millipore, MA, USA). The membranes were incubated with mouse monoclonal antibody against human FLOT1 (Sigma, St Louis, MO, USA) followed by horseradish peroxidase (HRP)-conjugated goat-anti-mouse IgG (Abcam), and the bands were detected using the Supersignal West Pico ECL chemiluminescence kit (Pierce) and Kodak X-ray film (Eastman Kodak Co, NY, USA); an anti-tubulin antibody (Sigma, St Louis, MO, USA) was used as a protein loading control.

### Statistical analysis

All experiments were performed at least three times, and all samples were tested in triplicate. The data are shown as the mean ± SEM unless otherwise noted; Student’s t-test was used for statistical analysis when only two groups were tested. A one-way analysis of variance was used to compare multiple groups. The difference in miR-124 and FLOT1 expressions between breast cancer specimens and normal adjacent tissues of human subjects was calculated by a two-tailed independent samples t-test. Spearman’s correlation analysis was used to determine the correlation between *miR-124* and FLOT1 expressions. In all cases, a *P* < 0.05 was considered statistically significant.

## Competing interests

The authors declared that they have no competing interests.

## Authors’ contributions

LM designed the experiment, interpreted the data and prepared the manuscript. LSL, JML and BW conducted the experiment, collected the data and helped to prepare the manuscript. DW, XHX, LJY, JLG, SYX, JG, XTL, YNK, XDX, HLT and XMX interpreted the data. All authors read and approved the final manuscript.

## References

[B1] Chaffer CL Weinberg RA A perspective on cancer cell metastasis Science 2011 12 1559 1564 10.1126/science.1203543 21436443

[B2] Bartel DP MicroRNAs: genomics, biogenesis, mechanism, and function Cell 2004 12 281 297 10.1016/S0092-8674(04)00045-5 14744438

[B3] Kasinski AL Slack FJ Epigenetics and genetics. MicroRNAs en route to the clinic: progress in validating and targeting microRNAs for cancer therapy Nat Rev Cancer 2011 12 849 864 10.1038/nrc3166 22113163 PMC4314215

[B4] Chen CZ MicroRNAs as oncogenes and tumor suppressors N Engl J Med 2005 12 1768 1771 10.1056/NEJMp058190 16251533

[B5] Croce CM Causes and consequences of microRNA dysregulation in cancer Nat Rev Genet 2009 12 704 714 10.1038/nrg2634 19763153 PMC3467096

[B6] Garzon R Marcucci G Potential of microRNAs for cancer diagnostics, prognostication and therapy Curr Opin Oncol 2012 12 655 659 10.1097/CCO.0b013e328358522c 23079782

[B7] Hoshino I Matsubara H MicroRNAs in cancer diagnosis and therapy: from bench to bedside Surg Today 2013 12 467 478 10.1007/s00595-012-0392-5 23129027

[B8] Cho WC MicroRNAs as therapeutic targets and their potential applications in cancer therapy Expert Opin Ther Targets 2012 12 747 759 10.1517/14728222.2012.696102 22690697

[B9] Jones CI Zabolotskaya MV King AJ Stewart HJS Horne GA Chevassut TJ Newbury SF Identification of circulating microRNAs as diagnostic biomarkers for use in multiple myeloma Br J Cancer 2012 12 1987 1996 10.1038/bjc.2012.525 23169280 PMC3516695

[B10] Zhang X Zeng J Zhou M Li B Zhang Y Huang T Wang L Jia J Chen C The tumor suppressive role of miRNA-370 by targeting FoxM1 in acute myeloid leukemia Mol Cancer 2012 12 56 10.1186/1476-4598-11-56 22900969 PMC3533721

[B11] Volinia S Galasso M Sana ME Wise TF Palatini J Huebner K Croce CM Breast cancer signatures for invasiveness and prognosis defined by deep sequencing of microRNA Proc Natl Acad Sci USA 2012 12 3024 3029 10.1073/pnas.1200010109 22315424 PMC3286983

[B12] Sieuwerts AM Mostert B Bolt-de Vries J Peeters D de Jongh FE Stouthard JM Dirix LY van Dam PA Van Galen A de Weerd V mRNA and microRNA expression profiles in circulating tumor cells and primary tumors of metastatic breast cancer patients Clin Cancer Res 2011 12 3600 3618 10.1158/1078-0432.CCR-11-0255 21505063

[B13] Hannafon BN Sebastiani P De Las Morenas A Lu JN Rosenberg CL Expression of microRNA and their gene targets are dysregulated in preinvasive breast cancer Breast Cancer Res 2011 12 R24 10.1186/bcr2839 21375733 PMC3219184

[B14] Lee MR Kim JS Kim KS miR-124a Is Important for migratory cell fate transition during gastrulation of human embryonic stem cells Stem Cells 2010 12 1550 1559 10.1002/stem.490 20665740

[B15] Cheng LC Pastrana E Tavazoie M Doetsch F miR-124 regulates adult neurogenesis in the subventricular zone stem cell niche Nat Neurosci 2009 12 399 408 10.1038/nn.2294 19287386 PMC2766245

[B16] Han ZB Yang Z Chi Y Zhang L Wang Y Ji Y Wang J Zhao H Han ZC MicroRNA-124 suppresses breast cancer cell growth and motility by targeting CD151 Cell Physiol Biochem 2013 12 823 832 10.1159/000350100 23816858

[B17] Wang P Chen L Zhang J Chen H Fan J Wang K Luo J Chen Z Meng Z Liu L Methylation-mediated silencing of the miR-124 genes facilitates pancreatic cancer progression and metastasis by targeting Rac1 Oncogene 2013 doi: 10.1038/onc.2012.598 10.1038/onc.2012.598 23334332

[B18] Xia JT Wu ZQ Yu CP He WL Zheng HQ He YL Jian WH Chen LZ Zhang LJ Li W miR-124 inhibits cell proliferation in gastric cancer through down-regulation of SPHK1 J Pathol 2012 12 470 480 10.1002/path.4030 22450659

[B19] Zheng F Liao YJ Cai MY Liu YH Liu TH Chen SP Bian XW Guan XY Lin MC Zeng YX The putative tumour suppressor microRNA-124 modulates hepatocellular carcinoma cell aggressiveness by repressing ROCK2 and EZH2 Gut 2012 12 278 289 10.1136/gut.2011.239145 21672940

[B20] Shi XB Xue L Ma AH Tepper CG Gandour-Edwards R Kung HJ Devere White RW Tumor suppressive miR-124 targets androgen receptor and inhibits proliferation of prostate cancer cells Oncogene 2013 12 4130 4138 10.1038/onc.2012.425 23069658 PMC4111479

[B21] Lv XB Jiao Y Qing Y Hu H Cui X Lin T Song E Yu F miR-124 suppresses multiple steps of breast cancer metastasis by targeting a cohort of pro-metastatic genes in vitro Chin J Cancer 2011 12 821 830 10.5732/cjc.011.10289 22085528 PMC4013330

[B22] Liang YJ Wang QY Zhou CX Yin QQ He M Yu XT Cao DX Chen GQ He JR Zhao Q MiR-124 targets Slug to regulate epithelial-mesenchymal transition and metastasis of breast cancer Carcinogenesis 2013 12 713 722 10.1093/carcin/bgs383 23250910 PMC3581604

[B23] Xiong P Xiao LY Yang R Guo Q Zhao YQ Li W Sun Y Flotillin-1 promotes cell growth and metastasis in oral squamous cell carcinoma Neoplasma 2013 12 395 405 10.4149/neo_2013_051 23581411

[B24] Zhang PF Zeng GQ Hu R Li C Yi H Li MY Li XH Qu JQ Wan XX He QY Identification of flotillin-1 as a novel biomarker for lymph node metastasis and prognosis of lung adenocarcinoma by quantitative plasma membrane proteome analysis J Proteomics 2012 12 202 214 22982323 10.1016/j.jprot.2012.08.021

[B25] Song L Gong H Lin C Wang C Liu L Wu J Li M Li J Flotillin-1 promotes tumor necrosis factor-alpha receptor signaling and activation of NF-kappaB in esophageal squamous cell carcinoma cells Gastroenterology 2012 12 995 1005 e1012 10.1053/j.gastro.2012.06.033 22732732

[B26] Lin C Wu Z Lin X Yu C Shi T Zeng Y Wang X Li J Song L Knockdown of FLOT1 impairs cell proliferation and tumorigenicity in breast cancer through upregulation of FOXO3a Clin Cancer Res 2011 12 3089 3099 10.1158/1078-0432.CCR-10-3068 21447726

[B27] Gong H Song L Lin C Liu A Lin X Wu J Li M Li J Downregulation of miR-138 sustains NF-kappaB activation and promotes lipid raft formation in esophageal squamous cell carcinoma Clin Cancer Res 2013 12 1083 1093 10.1158/1078-0432.CCR-12-3169 23319823

[B28] Iorio MV Croce CM microRNA involvement in human cancer Carcinogenesis 2012 12 1126 1133 10.1093/carcin/bgs140 22491715 PMC3514864

[B29] Bartel DP MicroRNAs: target recognition and regulatory functions Cell 2009 12 215 233 10.1016/j.cell.2009.01.002 19167326 PMC3794896

[B30] van Kouwenhove M Kedde M Agami R MicroRNA regulation by RNA-binding proteins and its implications for cancer Nat Rev Cancer 2011 12 644 656 10.1038/nrc3107 21822212

[B31] Wilting SM van Boerdonk RA Henken FE Meijer CJ Diosdado B Meijer GA le Sage C Agami R Snijders PJ Steenbergen RD Methylation-mediated silencing and tumour suppressive function of hsa-miR-124 in cervical cancer Mol Cancer 2010 12 167 20579385 10.1186/1476-4598-9-167 PMC2917428

[B32] Furuta M Kozaki KI Tanaka S Arii S Imoto I Inazawa J miR-124 and miR-203 are epigenetically silenced tumor-suppressive microRNAs in hepatocellular carcinoma Carcinogenesis 2010 12 766 776 10.1093/carcin/bgp250 19843643

[B33] Pierson J Hostager B Fan R Vibhakar R Regulation of cyclin dependent kinase 6 by microRNA 124 in medulloblastoma J Neurooncol 2008 12 1 7 10.1007/s11060-008-9624-3 18607543

[B34] Pinto R Pilato B Ottini L Lambo R Simone G Paradiso A Tommasi S Different methylation and microRNA expression pattern in male and female familial breast cancer J Cell Physiol 2013 12 1264 1269 10.1002/jcp.24281 23160909

[B35] Volinia S Croce CM Prognostic microRNA/mRNA signature from the integrated analysis of patients with invasive breast cancer Proc Natl Acad Sci USA 2013 12 7413 7417 10.1073/pnas.1304977110 23589849 PMC3645522

[B36] Chan M Liaw CS Ji SM Tan HH Wong CY Thike AA Tan PH Ho GH Lee AS Identification of circulating MicroRNA signatures for breast cancer detection Clin Cancer Res 2013 12 4477 4487 10.1158/1078-0432.CCR-12-3401 23797906

[B37] Cancer Genome Atlas Network Comprehensive molecular portraits of human breast tumours Nature 2012 12 61 70 10.1038/nature11412 23000897 PMC3465532

[B38] Gao J Li L Wu M Liu M Xie X Guo J Tang H MiR-26a inhibits proliferation and migration of breast cancer through repression of MCL-1 PLoS One 2013 12 e65138 10.1371/journal.pone.0065138 23750239 PMC3672200

[B39] Li LS Yuan LJ Luo JM Gao J Guo JL Xie XM MiR-34a inhibits proliferation and migration of breast cancer through down-regulation of Bcl-2 and SIRT1 Clin Exp Med 2013 12 109 117 22623155 10.1007/s10238-012-0186-5

[B40] Zhao Y Li Y Lou G Zhao L Xu Z Zhang Y He F MiR-137 targets estrogen-related receptor alpha and impairs the proliferative and migratory capacity of breast cancer cells PLoS One 2012 12 e39102 10.1371/journal.pone.0039102 22723937 PMC3377602

[B41] Rothe F Ignatiadis M Chaboteaux C Haibe-Kains B Kheddoumi N Majjaj S Badran B Fayyad-Kazan H Desmedt C Harris AL Global microRNA expression profiling identifies MiR-210 associated with tumor proliferation, invasion and poor clinical outcome in breast cancer PLoS One 2011 12 e20980 10.1371/journal.pone.0020980 21738599 PMC3126805

[B42] Cremona ML Matthies HJG Pau K Bowton E Speed N Lute BJ Anderson M Sen N Robertson SD Vaughan RA Flotillin-1 is essential for PKC-triggered endocytosis and membrane microdomain localization of DAT (vol 14, pg469, 2011) Nat Neurosci 2011 12 1617 1617 10.1038/nn.2781 PMC3066276 21399631

[B43] Singletary SE Allred C Ashley P Bassett LW Berry D Bland KI Borgen PI Clark GM Edge SB Hayes DF Staging system for breast cancer: revisions for the 6th edition of the AJCC cancer staging manual Surg Clin North Am 2003 12 803 819 10.1016/S0039-6109(03)00034-3 12875597

[B44] Li L Xie X Luo J Liu M Xi S Guo J Kong Y Wu M Gao J Xie Z Targeted expression of miR-34a using the T-VISA system suppresses breast cancer cell growth and invasion Mol Ther 2012 12 2326 2334 10.1038/mt.2012.201 23032974 PMC3519992

